# Occupational lead toxicity in battery workers

**DOI:** 10.12669/pjms.314.7066

**Published:** 2015

**Authors:** Shahla Basit, Nasim Karim, Alia Bano Munshi

**Affiliations:** 1 Shahla Basit, MBBS Principal Medical Officer; 2 Nasim Karim, FCPS.PHD Professor and Head Pharmacology Department, Bahria University Medical & Dental College, Karachi, Pakistan; 3 Alia Bano Munshi, PHD Chief Scientific officer, 1&3 PCSIR, Karachi

**Keywords:** Occupational Health, Lead Exposure, Lead Toxicity, Blood Lead Level, Atomic Absorption spectrophotometer

## Abstract

**Objective:**

To estimate blood lead level (BLL) and to assess the features of lead toxicity among lead acid battery (LAB) industrial workers.

**Methods:**

This prospective study was carried out in the medical centre of PCSIR Laboratories, Karachi from March 2012 – March 2013. Fifty LAB industry workers, males and females between 17-65 years were included in the study. They were divided into group 1 with 40 workers- directly related and group 2 with 10 workers –indirectly related, (administration staff members) to LAB manufacture. Detailed history, complete physical examination and BLL estimation by flameless atomic absorption spectrophotometer was done.

**Results:**

Total 34 patients in group 1 and 3 patients in group 2 had elevated BLL. Comparison of group 1 and 2 revealed anemia (40v/s4), bone pain (38v/s7), abdominal pain (38 v/s 2), nausea (32v/s6), head ache & irritability (24v/s6), weakness & lethargy (21v/s6), tremors (13v/s0) insomnia (5v/s4), lead line (4v/s 0) and blood pressure above 140/90 mm of Hg (12v/s0) respectively.

**Conclusion:**

High blood lead level and features attributable to lead toxicity were prevalent among LAB industry workers of Karachi.

## INTRODUCTION

Lead is a chemical element in the carbon group with symbol Pb.^1^ Lead has been used for thousands of years in lead acid batteries, bullets and shots, as a radiation shield^2^ and is recognized as an environmental and occupational pollutant.^3^-^5^ Adults are mainly exposed to lead at their workplaces through inhalation of lead laden particles, poor personal hygiene, water and food also contribute to the exposure.^6^ Lead poisoning has been documented since the ancient times of Greece and China.^7^

The US Occupational Safety and Health Administration (OSHA) lead standards require workers to be removed from lead exposure when their blood lead level is equal or above 60 μg/dL (general industry). Workers may return to work only when the BLL is below 40 μg/dL.^8^,^9^

Anemia is the classic manifestation of lead toxicity. Lead exposure reduces the lifespan of the erythrocytes and inhibits the heme biosynthesis. “Lead line” on the gum is often associated with other common gastrointestinal manifestations like abdominal colic, nausea, and vomiting. Chronic low lead exposure has been associated with elevation in blood pressure.^4^,^9^

In Pakistan, use of LAB has sharply risen because of enhanced demand in the transport sector, presenting a number of challenges related to local human health, regional ecosystem health, global climate change, urban livability, fiscal stability and energy security.^10^ About 95% lead acid batteries in Pakistan are manufactured by recycling batteries and scrap metal.^11^

It is documented that prevalence of Lead toxicity is extremely common in LAB workers. Health of the LAB workers is very much neglected. There is ample chance of exposure to lead among the workers present in an environment polluted by lead. This is predisposing them to develop lead toxicity.^12^

Present study was undertaken to estimate the blood lead level and to assess the features attributable to lead toxicity among lead acid battery industrial workers in Karachi.

## METHODS

This descriptive study was carried out at the medical center of PCSIR Karachi, for a period of one year, following approval by Ethical Review Board (ERB) of PCSIR. A total of 50 LAB workers referred by the factory doctor to the PCSIR medical center upon being unable to perform their duties were included in this study. They were interviewed, examined by PCSIR doctors and subjected to estimation of BLL, following an informed consent. A structured questionnaire regarding demographic data was recorded. 3-4mL blood sample was collected in EDTA tube for estimation of BLL.

### Grouping

Subjects were divided into two groups, (1) Battery workers involved directly with LAB manufacturing, 40 in number (2) Non Battery workers, who were indirectly involved, 10 in number.

### Statistical Analysis

Data was analyzed by SPSS version 16. P value of 0.05 or less was considered statistically significant for the results.

## RESULTS

The mean age of workers in group 1 was 35.4±15.4 years and majority (18) workers belonged to the age group of 36-55 years. While in group 2 (5) 50% workers were in the age group of 26-35 years as no female in group 1 and 3 females in group 2. Amongst the battery workers 45% were matric pass and 15 % were illiterate and only 8% received high school education. In group 2 all the workers were literate with 20% matric passed and 80% above matric. (Table-I)

**Table-I:** Demography & Educational Characteristics Battery Workers V/S non Battery Workers. N=50

S. No	Factors	Battery Workers n=40	Mean BLL ug/dL	St. dev.	Non Battery workers n=10	Mean BLL ug/dL	St. dev.
1	Age (Year)					
	17-25	9	73.33	29.46	2	12.50	14.85
	26-35	12	66.25	49.34	5	12.40	5.46
	36-55	18	58.33	29.64	2	14.00	9.90
	56-65	1	116.05	--	1	21.45	--
2	Gender					
	Male	40	60.00	37.73	07	18.37	13.32
	Female	0	--	--	03	7.33	3.50
3	Education					
	Illiterate	6 (15%)	48.67	34.50	0	--	--
	Under Matric	13 (33%)	59.62	40.67	0 (0%)	--	--
	Matric	18 (45%)	55.78	33.66	2 (20%)	16.5	7.78
	Above Matric	3 (8%)	53.33	6.11	8 (80%)	11	7.11

In group 1, 74% (15) workers had job duration of 10-15 years. The BLL showed an increase with the increase in the working hours per day. Most (55%) of them did not use any personal safety measures such as gloves, mask etc. A slight rise in BLL was seen in the workers who did not use PPE in comparison to those who took some measures. The working facilities were bad. There were 30% smokers in group 1. The overall working facilities provided to group 2 were a bit better than the group 1 but they were also not up to the standards. There were 60% smokers in group 2.(Table-II)

**Table-II:** Factors having impact on general health of Battery Workers. N=50.

S.#	Factors	Battery workers Frequency n=40	Mean BLL µg/dL	SD	Non battery workers Frequency n=10	Mean BLL µg/dL	SD
1	Year of Job less than 5 year	10	60	39.27	2	10	1.41
	5 to 10 years	9	70.33	42.97	8	9.37	7.78
	10 to 15 years	15	74.80	39.88	--		
	More than 15	6	90.67	38.92	--		
2	Duration of work (Hours)						
	8 hours	27	59.04	34.97	10	10.9	8.08
	10 hours	0	--	--	--		
	12 hours	12	61	40.65	--		
	16 hours	1	106	--	--		
3	Safety Measures						
	(i)Use of PPE						
	Yes	18	57.31	27.49	--		
	No	22	64.33	37.72	10	10.9	8.08
	(ii) General Room Dimension						
	Small	35	68.11	39.96	9	11.4	8.38
	Big	5	56	5.66	1	6.0	
	(iii) Lighting Facilitate						
	Good	0	--	--	7	8.85	6.91
	Insufficient	40	60	37.73	3	15.66	10.11
	(iv) Room Temperature						
	Comfortable	0	--	--	4	5.25	3.94
	Hot	40	60	37.73	6	14.66	8.11
	(v) Exhaust Fans						
	Present	2	51	12.73	1	4.23	
	Absent	38	61.32	38.34	9	11.88	7.99
	(vi) Air Conditioner						
	Present	0	--	--	1	7.0	
	Absent	40	60	37.73	9	11.33	8.45
4	Smoking Habits						
	Yes	12	72.67	44.99	6	15.5	7.25
	No	28	56.92	32.71	4	4.83	1.63
5	Shower Regularity						
	Yes	35	60.74	36.27	2	3.25	1.41
	No	5	66	43.17	8	12.87	7.84

The mean blood lead level of the workers in group1 was found to be 60.45± 14.54 μgm/dL. On the other hand, lead level was found to be less (15.47 μgm/dL) among the workers of group 2. 34 patients in group 1 and 3 patients in group 2 had raised BLL (Table-III).

**Table-III:** Groups & Blood Lead Level. N=50

Groups	BLL	Range	Mean	St.dev.
Group 1	Normal 06	< 20 ug/dL	17.61	1.91
Battery workers n=40	Raised 34	25-148 ug/dL	69.17	37.68
Group 2	Normal 07	< 20 ug/dL	12.17	1.76
Non Battery workers n=10	Raised 03	25-55 ug/dL	19.30	1.23

The subjects had anemia, headache, abdominal pain, nausea, tremors etc. and had higher mean BLL than those who did not had such illnesses. Hemoglobin (Hb) level of the workers ranged from 4.75 to 12.80 gm/dL and the mean Hb level was 8.77 ±(0.747)gm/dL. Out of the total 40 examined blood samples, 40 (100%) workers were found to be anemic and had lower Hb levels and higher mean BLL. While in group 2 (n=10) only 40% workers had anemia, with mean BLL of 23.75 ug/dL which is much lower than the workers of group 1, having anemia with BLL 60ug/dL. The blood pressure in 30.25% workers was found to be raised (BP >140/90 mmHg) with higher BLL of 71.67. In group 2 none of the participants were hypertensive. Another symptom extremely common in battery workers was abdominal pain. In group 1 (n=40) 95% workers had abdominal pain while in group 2 (n=10) only 20% had this symptom. The mean BLL of workers having abdominal pain in group 1 was 62.66ug/dL while in group 2 it was 35ug/dL. Bone pain was present in both groups in majority of the subject along with the weakness, lethargy and irritability. (Table-IV)

**Table-IV:** Signs & Symptoms Attributable to Lead Toxicity. N=50

S. No	Signs & symptoms	Battery workers n=40	Mean BLLug/dL	SD	P-value	Non battery workers n=10	Mean BLLug/dL	SD	P-value
1	Abdominal pain							
	Yes	38	62.66	35.61	0.195	02	35	14.14	0.247
	No	02	29	12.73		08	10.25	5.00	
2	Nausea							
	Yes	32	64.38	40.75	0.022*	06	20.66	13.36	0.067
	No	08	42.38	15.93		04	8.00	3.16	
3	Anemia							
	Yes	40	60	37.73	NA	04	23.75	16.83	0.170
	No	0	--	--		06	8.5	2.88	
4	Bone pain							
	Yes	38	61.42	35.90	0.409	07	18.14	13.83	0.063
	No	02	40	2.83		03	6.33	2.08	
5	Weakness & lethargy							
	Yes	21	65.71	40.22	0.298	06	19.16	13.8	0.103
	No	19	53.79	29.90		04	8.00	2.94	
6	Headache & irritability							
	Yes	24	63.83	37.86	0.476	06	15.66	7.94	0.069
	No	16	55.19	36.22		04	7.00	2.16	
7	Tremors							
	Yes	13	70.77	43.61	0.283	0	--	--	NA
	No	27	57.22	33.32		10	15.2	12.2	
8	Insomnia							
	Yes	05	72.80	39.33	0.394	04	25.75	13.93	0.087
	No	35	58.03	35.46		06	8.16	2.48	
9	Lead Line							
	Yes	04	108	36.22	0.009*	0	--	--	NA
	No	36	57.22	35.45		10	15.2	12.2	
10	Blood pressure							
	Below 140/90	28	57.14	32.73	0.260	10	15.2	12.2	NA
	Above 140/90	12	71.67	45.42		0	--	--	

* = Statistically significant.

## DISCUSSION

Occupational lead toxicity especially in the profession of battery manufacturing is extremely common worldwide similar is the situation in Pakistan. The subjects were referred from leading battery manufacturing companies of Karachi, situated in industrial areas however even in these leading companies the working conditions were very poor. The battery workers were mostly middle aged in group 1 that is 36-55 years old. It was noted that the symptoms were severe in young patients (17-25 years) in spite of the fact that the duration of their job in the factory was much less than those of the middle aged workers. The children are more vulnerable to lead and are more susceptible to develop lead toxicity, particularly neurological toxicity even at low level of exposure.^13^,^14^ It is also documented that the facilities of ventilation, comfortable room temperature, good lighting and big space of working helps in reduction of lead absorption.^15^ However in group 2 the younger age group (17-25 years) exhibited low BLL in comparison to the old groups probably on account of indirect exposure.

In group 1, 46% of the workers were either primary passed or illiterate. Considering the present age of the workers and their years of job, it is evident that most of the workers were exposed to lead continuously for a long time. Studies carried out in India^16^ and in Bangladesh^17^ have also depicted similar picture of the working conditions and the level of education favoring our findings.

About 30% workers in group 1 and 60% in group 2 were smokers. The BLL was found 14.32 μgm/dL higher among smokers. This finding is consistent with other study findings, which revealed that smoking at workplace is significantly related to BLL concentration and is found to be higher among the smokers than nonsmokers.^18^

According to OSHA, the BLL should be less than 40 μgm/dL and if it is more than 40 μgm/dL, the worker must be notified in writing and provided with medical examination. The workers may return to the job only if two consecutive BLL are less than 40 μgm/dL.^8^,^9^ Therefore, it could be apprehended that the workers of these industries were chronically exposed to lead and were developing lead toxicity.

Inhalation of lead might also occur by air borne lead particulate matter during melting to recover lead.^19^,^20^ Lead absorption from lungs depends upon vapor versus particle size and concentration of the metal present. About 90% of lead particles are small and are readily absorbed through alveoli into blood.

Chronic lead toxicity has effect on hematopoietic system, gastrointestinal system, renal, reproductive and nervous system. Anemia results due to impairment of heme synthesis and acceleration of red blood destruction.^20^ Moreover, it was found that the mean BLL was high among the workers who had anemia. Increased blood lead concentration can also reduce serum ferritin and body iron levels besides HB% and hematocrit values.

Prolonged high lead exposure was reported to cause elevated blood pressure. The association between BLL and BP. depends on multiple factors like inactivation of endogenous nitric oxide, alteration in renin-angiotensin-aldosterone system, changes in calcium ions in vascular smooth muscles, rise in endothelin and thromboxane levels etc.

High risk of lead toxicity among Karachi battery workers is consistent with studies of battery workers in other developing countries.^21^ Only 4 (8%) workers had lead line in our study with highest BLL levels in comparison to all other participants. Similar findings have been documented in a study where 28% workers had lead line and BLL were highest in comparison to those without lead line.^21^ A key role for health agencies, besides providing opportunities for diagnosis and therapy, should be to increase public awareness about this widespread environmental hazard through education, documentation and communication.

## CONCLUSION

High blood lead level and illnesses attributable to lead toxicity were prevalent among LAB industry workers whether they were directly or indirectly involved in the manufacturing of LAB. Taking measures related to occupational health as hygiene and safety at the work place can improve the existing poor working environment and prevent the lead toxicity. This in turn might be able to reduce the morbidity and mortality along with the economic loss to our country.
